# Filtrating colorectal cancer associated genes by integrated analyses of global DNA methylation and hydroxymethylation in cancer and normal tissue

**DOI:** 10.1038/srep31826

**Published:** 2016-08-22

**Authors:** Ming Li, Fei Gao, Yudong Xia, Yi Tang, Wei Zhao, Congcong Jin, Huijuan Luo, Junwen Wang, Qingshu Li, Yalan Wang

**Affiliations:** 1 Department of Pathology, Molecular Medicine and Cancer Research Center, Chongqing Medical University, 1 Yixueyuan Road, Yuzhong District, Chongqing 400016, China; 2 BGI-Shenzhen, Build 11, Beishan Industrial Zone, Yantian District, Shenzhen 518083, China; 3 Agricultural Genomes Institute at Shenzhen, Chinese Academy of Agricultural Sciences, 7 Pengfei Road, Dapeng New District, ShenZhen 518118, China; 4 E-GENE Technologies, Co., Ltd., 16 West Jinniu Road, Pingshan New District, Shenzhen, 518118, China

## Abstract

Recently, 5-hydroxymethylcytosine patterning across the tumor genome was considered as a hallmark of cancer development and progression. However, locus-specific difference of hydroxymethylation between colorectal cancer and normal tissue is unknown. In this study, we performed a newly developed method, HMST-seq, to profile 726 aberrant methylated loci and 689 aberrant hydroxymethylated loci synchronously in genome wide of colorectal cancers, majority of which presented higher methylation or lower hydroxymethylationin than in normal group. Besides, abnormal hydroxymethylated modification was more frequently occur at proximal regions close to TSSs and TSSs regions than abnormal methylation. Subsequently, we screened four genes (ALOX15, GHRHR, TFPI2 and TKTL1) with aberrant methylation and aberrant hydroxymethylation at some genome position by functional enrichment analysis as candidate genes associated with colorectal cancer. Our results may allow us to select differentially epigenetically modified target genes implicated in colorectal cancer tumorigenesis.

DNA methylation is an important epigenetic modification for gene transcription, and an amount of evidence has been shown that genes with high levels of 5-methylcytosine (5mC) in their promoter region are transcriptionally silent, subsequently, DNA methylation gradually accumulates resulting in long-term gene silencing. Aberrant DNA methylation patterns – hyper-methylation and hypo-methylation differ from normal tissue – have been discovered in many kinds of human cancers^1^,^2^. Hyper-methylation typically occurs at CpG islands in the promoter region and is associated with gene inactivation. Global hypo-methylation has also been implicated in the progression of cancer through different mechanisms. Typically, there is hyper-methylation of tumor suppressor genes (TSGs) and hypomethylation of oncogenes.

5-mC can be oxidized to 5-hydroxymethylcytosine (5hmC), then, can be further oxidized to 5-formylcytosine (5fC) and 5- carboxylcytosine (5caC) by the ten-eleven translocation (TET) enzyme family proteins, which may play a role in DNA demethylation^3^,^4^. 5hmC has been found significantly reduced in malignant cells, including colorectal cancer (CRC)^5^. Since conventional technologies, such as bisulfite sequencing and restriction enzyme cutting technology, could not distinguish between 5-mC and 5-hmC^6^,^7^, and the existence of 5-hmC in samples could influence the accuracy of DNA methylation detection^8^. Thus, there is an urgent requirement of a new technology to detect 5-mC and 5-hmC simultaneously in malignant tumor genomic profile research for higher accuracy of detection of aberrant DNA methylation patterns compare to normal tissue.

In this research, we used a newly developed single-base high-throughput sequencing approach (hydroxymethylation and methylation sensitive tag sequencing (HMST-seq)) to globally detect distribution of both 5mC and 5hmC at CCGG sites synchronously, in CRC samples and their adjacent non-cancerous normal tissues. Subsequently, we performed functional analysis of genes containing differentially hydroxymethylated or methylated regions (DhMR or DMR) by **WEB**-based **GE**ne **S**e**T A**na**L**ysis **T**oolkit (WebGestalt) to screen genes associated with colorectal cancer. As a result, four genes, including ALOX15, GHRHR, TFPI2 and TKTL1, were revealed as key CRC-associated candidate genes with aberrant methylation and aberrant hydroxymethylation at same promoter position. Significant hypermethylation as well as hypo-hydroxymethylation of promoters were revealed for these genes in CRC samples. In conclusion, based on novel HMST-seq technology, our study identified new candidate genes that were epigenetically modulated in CRC, which can be applied as clinical biomarkers or candidates of tumor suppressor genes in future studies.

## Results

### Distribution of 5mC and 5hmC in CRC samples and matching normal tissues

To investigate the distribution of 5mC and 5hmC in CRC specimens and matching normal samples, a new improved technology ‘hydroxymethylation and methylation sensitive tag sequencing (HMST-seq)’ was performed on the 16 pairs of CRCs and accordingly normal samples. 5mC and 5hmC can be investigated using HMST-seq at ‘CCGG’ sites with a single-base resolution, and three libraries were established for every sample, including ‘C + mC + hmC’ library represented to contain unmodified C, mC, and hmC tags through MspI-mediated digestion of genomic DNA, ‘C + mC’ library represented to contain C and mC tags through MspI-mediated digestion of glucosylated genomic DNA and ‘C’ library represented to contain C tags only through HpaII-mediated digestion of normal genomic DNA^9^. GRSN method was performed to normalize the data for the three libraries.

**Figure 1:**
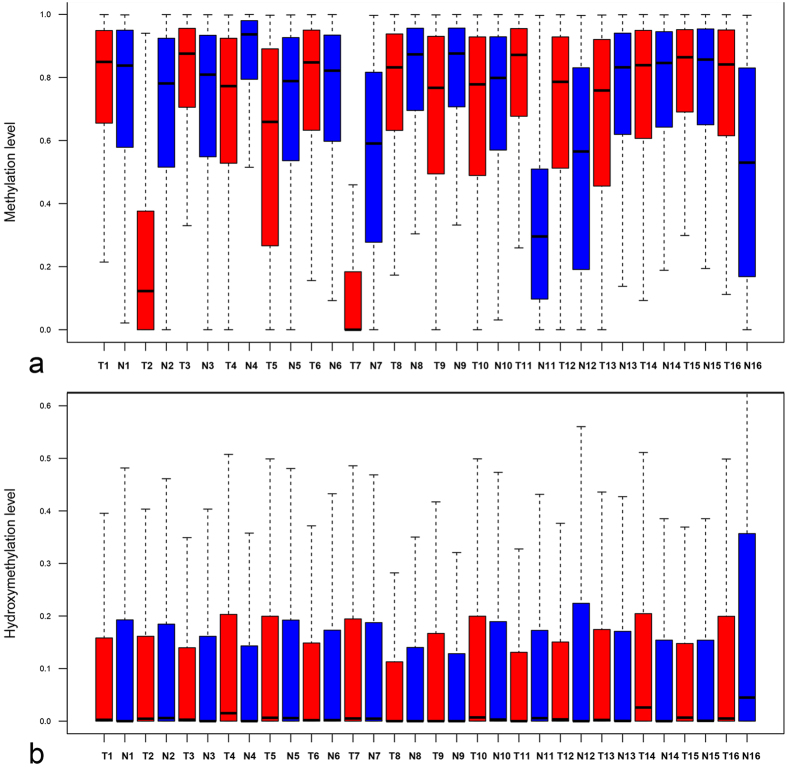
Boxplot showing distribution of methylation and hydroxymethylation levels of each detectable CCGG sites in genome wide of CRCs (red) and matched normal sample (bule). The hydroxymethylation level is defined as the ratio between ‘C + mC + hmC’ and ‘C + mC’ tags, while methylation abundance is defined as the ratio between tag counts of “C + mC” and “C” libraries. T1 is a sign of patient 1’s tumor sample, while N1 is a sign of patient 1’s normal sample.

On average, approximately 342 million reads for each library were generated, and about 110.7 million (32.34%) reads were aligned to a virtual library of the human genome (hg19). An average of 57.23% uniquely aligned reads (0 mismatch + 1 mismatch) and 25.59% multiple aligned reads (0 mismatch + 1 mismatch) were then obtained for each library, resulting in an average of 1.346 million total cytosine with greater than 30 X sequencing depth for each library (see Supplementary Table S1). Based on our method, the hydroxymethylation level can be defined as the ratio between ‘C + mC + hmC’ and ‘C + mC’ tags, while methylation abundance can be represented by ratio between tag counts of “C + mC” and “C” libraries. We then examined the genome-wide distribution of methylation and hydroxymethylation levels of each detectable CCGG site. The majority of the CCGG sites displayed high level of methylation. In contrast, majority of the CCGG sites displayed low level of hydroxymethylation (Fig. 1). Thereby, on average, 1,162,336 (82,59%) and 106,907 (7.58%) total examined CCGG sites were identified as significant for 5mC and 5hmC, respectively, in matching normal specimens, while 5mC (1,105,904; 78.38%) and 5hmC (75,236; 5.33%) modification on informative sites were observed in the CRC specimens (see Supplementary Table S2) (sequencing depth >10 X, FDR < 0.001)^9^.

### DMRs and DhMRs were revealed by comparison of CRC and matching normal samples

**Figure 2:**
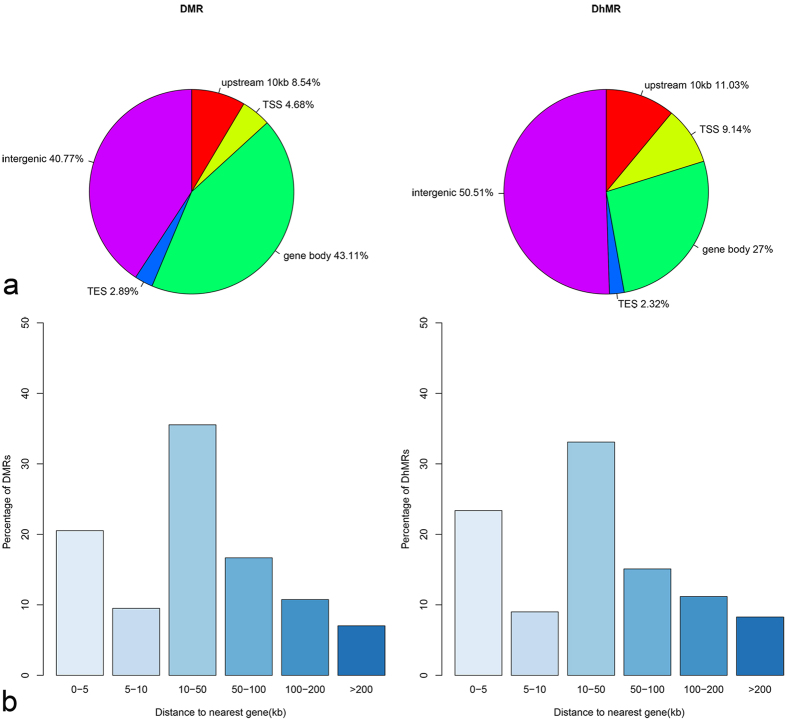
The distribution of DMRs and DhMRs. (**a**) The distribution ratio of DMRs and DhMRs within genomic regions. The DhMR or DMR located in two different elements are attributed to the element that overlaps with a bigger proportion of the DhMR or DMR and counted only once. (**b**) The distance of DMRs and DhMRs to transcriptional start sites (TSSs).

To identify differentially methylated regions (DMRs) and differentially hydroxymethylated regions (DhMRs) between CRC and matching normal samples according to the data of global methylation and hydroxymethylation levels of each detectable CCGG site in 16 pairs of CRC and non-CRC samples, a sliding window strategy was performed as our previous study^9^. Take the first 5 CCGG sites that contains at least 4 CCGG sites with the same changing trend between tumor and normal group, and Wilcoxon rank sum test P-value < 0.05 as the seed sites of a candidate DMR or DhMR. We found 726 DMRs (see Supplementary Table S3) and 689 DhMRs (see Supplementary Table S4) between CRCs group and normal tissue group, interestingly, we discovered that DhMRs were more likely to occur at proximal regions close to TSSs and TSSs regions (20.17%) rather than DMRs (13.22%), although most part of DMRs (83.88%) and DhMRs (77.51%) distributing in intergenic and gene body (Fig. 2).

**Figure 3:**
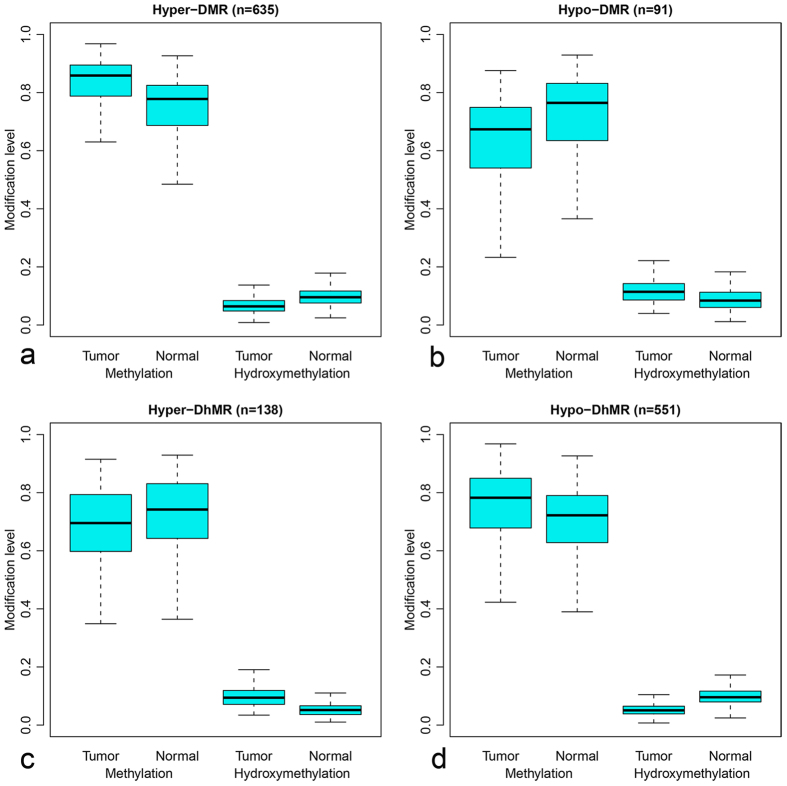
Range of methylation and hydroxymethylation level in DMRs and DhMRs. The vertical axis shows the abundance of methylation (left) and hydroxymethylation (right) in DMRs or DhMRs. Hyper-DMR (n = 635), loci with higher methylation in CRC group; Hypo-DMR (n = 91), loci with lower methylation in CRC group; Hyper-DhMR (n = 138), loci with higher hydroxymethylation in CRC group; Hypo-DhMR (n = 551), loci with lower hydroxymethylated modification in CRC group; all of these DMRs and DhMRs are confirmed by statistics analysis comparing with normal tissue.

635 out of 726 DMRs (87.47%) were hypermethylated, while 551 out of 689 DhMRs (79.97%) were hypo-hydroxymethylated in CRC group (Fig. 3). Considering the facts that 5hmC is an intermediate during in TET-associated DNA demethylation, which is one of proposed mechanisms of DNA demethylation^10^, lower level of hydroxymethylation suggested for less dynamic preprogramming rate for specific genomic region. All together, the high percentage of hypermethylated DMRs as well as hypo-hydroxymethylated DhMRs both indicated that majority of detected CCGG sites by HMST-seq were stably hypermethylatd in CRC group comparing with normal controls. Nevertheless, only 114 genes were detected containing both DMR and DhMR, among which only 38 genes were with DMR and DhMR at the same location, at least partly supporting the role of 5-hmC as an inter-mediate in DNA demethylation. Among 38 genes with DMR and DhMR at same genome position, 35 genes were detected hyper-methylation and hypo-hydroxymethylation in CRC group, and 11 out of these 35 genes containing DMR and DhMR at promoter region.

### CRC-associated candidate genes with aberrant DNA modifications

**Figure 4:**
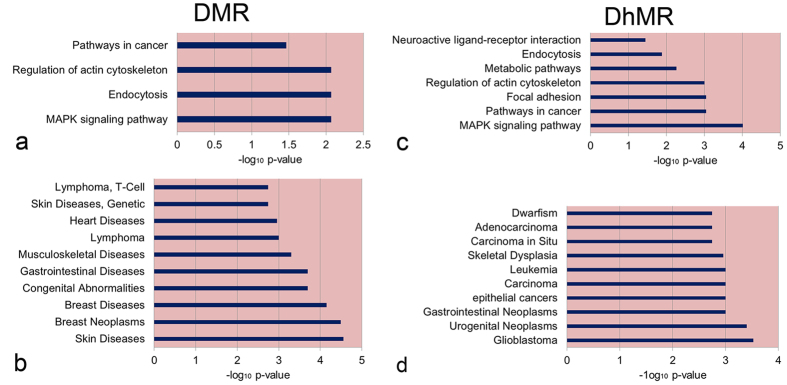
KEGG pathway and diseases enrichment analysis of genes related to DMRs or DhMRs. (**a**) KEGG pathway enrichment analysis of DMRs, (**b**) diseases enrichment analysis of DMRs, (**c**) KEGG pathway enrichment analysis of DhMRs, (**d**) diseases enrichment analysis of DhMRs. The x-axis shows the P value from hypergeometric test adjusted by the multiple test adjustment.

Functional enrichment analysis for genes with DMRs or DhMRs between CRC and matching normal samples was performed using WebGestalt. We found that DMR and DhMR genes were enriched in ‘Pathways in cancer’, ‘MAPK signaling pathway’, ‘Endocytosis’ and ‘Regulation of actin cytoskeleton’ in KEGG pathways, but these genes rarely overlapped. Besides, cancer-related pathways including, ‘Focal adhesion’, ‘Neuroactive ligand-receptor interaction’, and ‘Metabolic pathways’ were enriched for DhMR genes (see Supplementary Table S5 and Fig. 4). According to the disease enrichment analysis, we found that DhMR genes were notably enriched in epithelial carcinomas or gastrointestinal neoplasms such as, ‘Gastrointestinal Neoplasms’, ‘epithelial cancers’, ‘Carcinoma’, ‘Carcinoma *in Situ*’, and ‘Adenocarcinoma’, while only 11 DMR genes were detected associated with ‘Gastrointestinal Diseases’ (see Supplementary Table S6 and Fig. 4).

We then selected 53 DMR genes that significantly enriched in 10 disease categories and 2 KEGG categories (‘Pathways in cancer’ and ‘MAPK signaling pathway’), as well as 56 DhMR genes that significantly enriched in 10 disease categories and 3 KEGG categories (“Focal adhesion”, “Pathways in cancer” and “MAPK signaling pathway”) as candidate genes associated with colorectal cancer.

Among these 103 candidate genes, we found 6 genes were common in both DMR and DhMR data, including ALOX15, CCNE1, FGFR2, GHRHR, TFPI2 and TKTL1. Among these 6 genes, ALOX15, GHRHR, TFPI2 and TKTL1 contained both DMR and DhMR in the same genomic position. We then examined the modification status of these genes. Opposite tendency of methylation and hydroxymethylation changes was revealed for all these genes, that is, all genes were significantly hypermethylated as well as hypo-hydroxymethylated in CRC samples. As these DMRs or DhMRs were all distributed in promoter or TSS regions, these genes might be suppressored for gene expression in CRC.

## Discussion

Hyper-methylation is considered as the principle mechanism of dysfunction of tumor suppressor genes in cancer progression, moreover, promoter hyper-methylation status of some tumor suppressor genes in colon adenocarcinoma were confirmed by previous studies^11^,^12^, while hydroxymethylation has been reported as an epigenetic marker whose decrease is broadly and tightly associated with cancer development^13^,^14^,^15^. Since 5-hydroxymethylation in cancer research is still in infancy, further investigation is needed. Nevertheless, some conventional technologies cannot distinguish 5hmC from 5mC, such as bisulfite treatment or methylation-sensitive enzyme digestion-based methods^6^,^7^. Affinity enrichment assays are bulk assays and are unable to resolve 5mC with nucleotide precision^16^. Therefore, difference technologies might lead to conflicting results among close research studies, and impact inaccurate quantification of DNA methylation levels. Thus, a sensitive and specific technology is required to distinguishing between 5hmC and 5mC in the identification of DNA methylation.

In this study, we applied the newly developed HMST-Seq technology, which was confirmed as a cost-effective and selective detection method of epigenetic modification^9^,^17^, to profile DNA methylation and hydroxymethylation levels of CRC samples and matching normal tissue synchronously in genome wide. According to our data, 106,907 (7.58%) and 75,236 (5.33%) of the total CCGG sites were called out as 5hmC sites in control and CRC samples, respectively. If we used bisulfite-treatment based methods, but not HMST-seq, all of these 5hmC sites will be taken as 5mC, since conventional bisulfite-treatment based methods can’t differentiate 5hmC from 5mC^6^,^7^. This result clearly indicated for the increased sensitivity of HMST-seq in examining DNA modifications. Regarding to epigenetic changes of CRC genomes, our results showed 726 DMRs and 689 DhMRs between CRCs and normal samples, and most of they presented hyper-methylation or lower level of hydroxymethylation in CRC samples, indicating majority of detected CCGG sites by HMST-seq were stably hypermethylatd in CRCs comparing with normal controls. Among 38 genes with DMRs and DhMRs at same locus, 35 genes were detected with hyper-methylation and hypo-hydroxymethylation in CRC group, and 11 out of these 35 genes containing DMR and DhMR at promoter region, suggesting that reducing of 5hmC and increasing of 5mC in these 35 genes were observed at same genome position in CRCs comparing with normal tissue, moreover promoter was the most frequent location for observing changes of 5mC and 5hmC level in these loci. 5hmC was reported as a strong inhibitor of the DNA methyltransferase maintenance methylation reaction catalyzed by DNMT1^18^,^19^, leading to passive DNA demethylation over subsequent replication cycles. Thus, depletion of 5hmC of specific locus in CRC probably resulted in reduction of DNA methyltransferase inhibition, generating hyper-methylation at this locus. Thus, we inferred that low level of hydroxymethylation of promoters might mark or even facilitate DNA hypermethylation at same loci of CRCs by recovering activity of DNA methyltransferase. However, DNA methylation-demethylation is a complex story, and global changes in DNA methylation are thus likely to require a concerted action of several DNA demethylation mechanisms, thus, TET-associated DNA demethylation is not the only proposed mechanism of DNA demethylation, other factor could also lead to DNA demethylation (eg. the activity of Dnmt1 DNA methyltransferase)^10^. Therefore, changes of 5mC in locus could not alone account for changes of TET-catalyzed 5-hydroxymethylcytosine level. In our data, the rest observed loci without identical location with both DMR and DhMR might be explained by other DNA demethylation mechanisms. But the detail needed to be further studied.

In present research, we discovered that DhMRs were more likely to occur at proximal regions close to TSSs and TSSs regions (20.17%) rather than DMRs (13.22%), illustrating that changes in 5hmC of CRCs probably play an important role in gene transcription and expression. Recently, 5hmC was considered as an important factor in transcript process^20^,^21^, most importantly, gene-specific 5-hmC changes in human colon cancers are directly correlated with changes in gene expression^22^, thus, finding gene-specific 5hmC changes in CRC seems very important to screen different expression of genes in CRC and normal tissue.

Based on KEGG data, we discovered 53 DMR genes and 56 DhMR genes that enriched multiple highly interrelated cancer signaling pathways, including ‘Focal adhesion’, ‘Pathways in cancer’ and ‘MAPK signaling pathway’ as well as some diseases, such as ‘Gastrointestinal Diseases’ and ‘Gastrointestinal Neoplasms’, these 103 were genes selected as candidate genes associated with colorectal cancer. Among these 103 candidate genes, the identical locus of DMR and DhMR was discovered in ALOX15, GHRHR, TFPI2 and TKTL1 genes.

15-Lipoxygenase-1 (15-LOX-1, ALOX15) is an inducible and highly regulated enzyme in normal human cells^23^ that plays an important role in the production of lipid signaling mediators^24^. Some researches have proved down-regulation of ALOX15 in various human cancers (e.g., cancers of the colon^25^,^26^,^27^, breast^28^, lung^29^, and pancreas^30^, supporting the theory that ALOX15 is a tumor suppressor gene in these cancers. Zou *et al.* found that the ALOX15 promoter was methylated in colorectal cancer cells *in vitro* and in 36% (18/50) of colorectal cancer patients but virtually absent in the normal colons without history of colorectal cancer or polyps^31^. Consistently, in this study, ALOX15 was observed with hyper-methylation and hypo-hydroxymethylation at promoter in CRC samples comparing with matching normal tissue. However, Zou elucidated that ALOX15 promoter DNA methylation level had no significant correlation with its expression, and promoter demethylation failed to reestablish ALOX15 expression^31^. These data indicated that promoter methylation was not the only reason for suppression of ALOX15, otherwise involve multiple layers of repression mechanisms. Since presence of 5hmC in promoter would impact the level of transcription, our characterization of lower hydroxymethylated level in promoter of CRCs, provides a possible reason of down-regulation of ALOX15 in colon cancer. The validate work need to be done in the future study.

Growth-hormone-releasing hormone receptor (GHRHR) is dominantly expressed in the pituitary but it is also found in various cancer cell lines and tumors^32^. Combination of GHRH and GHRHR activates the mitogen-activated protein kinases pathway (MAPKs), which is involved in cell proliferation and differentiation^33^. Hohla *et al.* discovered strong expression of GHRHR in tubulovillous adenomas and colorectal cancers and absence in normal colonic mucosa^34^. In our present research, GHRHR promoter was detected hyper-methylated and hypo-hydroxymethylated in CRC samples. Nevertheless, we cannot exclude other mechanism of boost expression of GHRHR. Further studies on histone modification or transcriptional activator are needed to explain why hyper-methylation of GHRHR promoter presents strong protein expression.

Tissue factor pathway inhibitor 2 (TFPI2), a Kunitz-type serine proteinase inhibitor, The protein can inhibit a variety of serine proteases including factor VIIa/tissue factor, factor Xa, plasmin, trypsin, chymotryspin and plasma kallikrein. Hibi *et al.* detected TFPI2 methylation in the serum of colorectal cancer patients^35^, while after curative surgery methylation of TFPI2 no longer detected in the serum DNA, indicating that methylated TFPI2 in serum DNA was derived from colorectal cancer^36^. Furthermore, he found methylation of TFPI2 is frequently observed in colorectal cancer, suggesting TFPI2 may act as a tumor suppressor in colorectal carcinomas^37^. However, no research was involved in analysis of hydroxymethylated level of TFPI2 and location of hydroxymethylation modification. Our data showed higher methylation and lower hydroxymethylation levels of TFPI2 at TSS in CRC samples, providing the specific locus with aberrant hydroxymethylated level, which could be another reason of impacting transcription of TFPI2.

Transketolase-like 1 (TKTL1) is a transketolase that acts as a homodimer and catalyzes the conversion of sedoheptulose 7-phosphate and D-glyceraldehyde 3-phosphate to D-ribose 5-phosphate and D-xylulose 5-phosphate. This reaction links the pentose phosphate pathway with the glycolytic pathway. Previous studies have proven that TKTL1 was required for rapid cell growth and proliferation of human colon cancer cells^38^,^39^. Interestingly, Diaz-Moralli *et al.* discovered that a significant association of TKTL1 expression with lymph-node involvement while a significant decrease of TKTL1 expression associated with metastasis^40^, similar appearance has been observed in mutation of ras in CRC: the incidence of mutation increases from 7% to 60% starting early till late carcinomas, but decreases to 45% in invasive phrase, indicating an possibility that TKTL1 contributed in cell aggressiveness but not in metastasized cells like ras mutation^41^,^42^. Besides, Smith *et al.* proposed that TKTL1 could be up-regulated in response to hypo-methylation in head and neck cancer and lung cancer^43^. In this research, we detected hypo-methylation and hyper-hydroxymethylation at TSS of TKTL1 in CRC, provided another suggestion that increasing expression of TKTL1 in aggressive CRC probably due to not only hypo-methylation but also hyper-hydroxymethylation at TSS.

In summary, our study highlighted genes with aberrant methylation and hydroxymethylation in CRCs by HMST-seq, subsequently, enrichment analysis of these genes was performed using WebGestalt and 103 genes were identified as differentially epigenetically modified target genes implicated in colorectal cancer tumorigenesis. Among them, ALOX15, GHRHR, TFPI2 and TKTL1 were observed the identical locus of DMR and DhMR. However, expression and functions of select differentially epigenetically modified target genes implicated in colorectal cancer tumorigenesis were needed to validate in further research base on our DMR and DhMR data.

## Materials and Methods

### Patient samples

This study was conducted under the principles of the World Medical Association Helsinki agreement. Ethical approval was obtained from the Chongqing Medical University Ethics Committee. Informed consent was obtained for experimentation with human subjects. All the 16 pairs of specimens (tumors and relevant adjacent tissues) were obtained from patients who signed informed consent before surgery (see Supplementary Table 7S), and immediately frozen at −80 °C in liquid nitrogen container until DNA extraction. The CRC diagnosis was confirmed through pathological examination.

### DNA extraction

Genomic DNA was extracted from samples according to the instruction of DNeasy Blood and Tissue kit (Qiagen, Valencia, CA, USA), provided by the manufacturer.

### Data analysis by hydroxymethylation and methylation sensitive tag sequencing (HMST-Seq)

Genomic DNA, extracted by DNeasy Blood and Tissue kit, was detected by HMST-Seq and the libraries were constructed on the basis of our previous study^9^. Three independent libraries with initially different enzyme digestion were constructed for each sample, which were termed “C” library (c), “C + mC” library (a) and “C + mC + hmC” (b), respectively. For “C + mC” library construction, an aliquot of genomic DNA from each sample was first glucosylated by incubating DNA substrates with T4 β-glucosyltransferase (β-GT) (NEB). After glucosylation, the aliquot of glucosylated DNA was then digested with MspI (NEB). For the “C” or “C + mC + hmC” libraries, the aliquots of DNA without glucosylation were directly digested with HpaII or MspI (NEB), respectively. After digestion, the digested aliquots of DNA from all three libraries were ligated with biotinylated linker, fragmented by NlaIII, captured by streptavidin-conjugated beads, digested with MmeI to generate short sequence tags (16–17 bp), and ligated with sequencing linkers and amplified by PCR. Then, the purified tags were sequenced using Illumina HiSeq analyzer according to the manufacturer’s instructions.

Sequencing data will be mapped to reference genome. Only the uniquely mapped reads can be used for standard analysis and personal bioinformatics analysis.

### Data Filtering and Alignment

We extracted the 16–17 bp tags from sequencing reads after base calling, adapter removal and low-quality reads filtering. Then the virtual library was constructed as follows: The human genome sequence (hg19) was *in silico* digested by *Msp*I or *Nla*III, we defined the DNA sequences between the nearest *Nla*III sites around each *Msp*I site in both directions as the virtual library reference for mapping. Using the open-source programming language Perl, all the tags were mapped to the virtual library with no more than one mismatch, and the unambiguous mapped tags were used for further analysis.

The data normalization among libraries was performed according to Global Rank-invariant Set Normalization (GRSN) method^44^, and the hydroxymethylation or methylation abundance of CCGG site was determined as the ratio between tag counts of two different libraries. Thus, the hydroxymethylation level can be defined as the ratio between ‘C + mC + hmC’ and ‘C + mC’ tags, while methylation abundance can be represented by ratio between tag counts of “C + mC” and “C” libraries. Moreover, CCGG sites containing significantly different tag counts (sequencing depth >10X, FDR < 0.001) based on Poisson distribution, simultaneously, a ratio of tags between two libraries larger than 1 were defined to be significantly modified sites.

### Differentially methylated or hydroxymethylated regions

Differentially hydroxymethylated or methylated regions were defined in the following steps: a) take the first 5 CCGG sites that contains at least 4 CCGG sites with the same changing trend between tumor and normal group, and Wilcoxon rank sum test P-value < 0.05 as the seed sites of a candidate DMR; b) then a 3′ downstream adjacent CCGG with the same changing trend was incorporated with this candidate DMR. Up to 2000 bp inter-distance was allowed between the two adjacent CCGGs, Wilcoxon rank sum test was performed in the incorporated region; c) repeat these steps until Wilcoxon rank sum test P-value >= 0.05; d) the incorporated region were defined as a DhMR or DMR.

### Enrichment analysis of DMRs and DhMRs

Functional enrichment analysis for genes with DMRs or DhMRs was performed using WebGestalt^45^,^46^, which is freely accessible at http://www.webgestalt.org.

## Additional Information

**How to cite this article**: Li, M. *et al.* Filtrating colorectal cancer associated genes by integrated analyses of global DNA methylation and hydroxymethylation in cancer and normal tissue. *Sci. Rep.* **6**, 31826; doi: 10.1038/srep31826 (2016).

## Supplementary Material

Supplementary Information
